# Acromial physeal fracture in an adolescent: a case report and literature review

**DOI:** 10.1016/j.jseint.2023.10.002

**Published:** 2023-11-07

**Authors:** Diego Gonzalez-Morgado, Enrique Alberto Vargas Meouchi, Diego Soza Leiva, Carla Carbonell Rosell, Raquel Sevil Mayayo, Daniel Pacha-Vicente

**Affiliations:** a Orthopaedic Surgery Department, Hospital Universitari Vall d’Hebron, Universitat Autonoma de Barcelona, Barcelona, Spain; b Orthopaedic Surgery Department, Pediatric Orthopedics Unit, Hospital Universitari Vall d’Hebron, Universitat Autonoma de Barcelona, Barcelona, Spain

**Keywords:** Acromion, Physeal fracture, Children, Pediatric, Resorbable screw, Biodegradable screw

Fractures of the acromion are rare across all age groups, and very few cases have been reported in children and adolescents.^1^^,^^2^^,^^8^^,^^12^ Typically, these fractures are observed in conjunction with other shoulder injuries. Magnetic resonance imaging (MRI) plays a crucial role in evaluating the extent of the injury and guiding appropriate treatment. Neglecting to treat displaced fractures can result in reduced shoulder motion, impingement, rotator cuff tears, and persistent pain. This report presents the clinical and surgical management approach for a 13-year-old girl who presented with a displaced acromial physeal fracture. Additionally, we provide a comprehensive review of the existing literature on this type of injury in the pediatric population.

The patient and parents were informed that data concerning the case would be submitted for publication and they agreed.

## Case report

**Figure 1:**
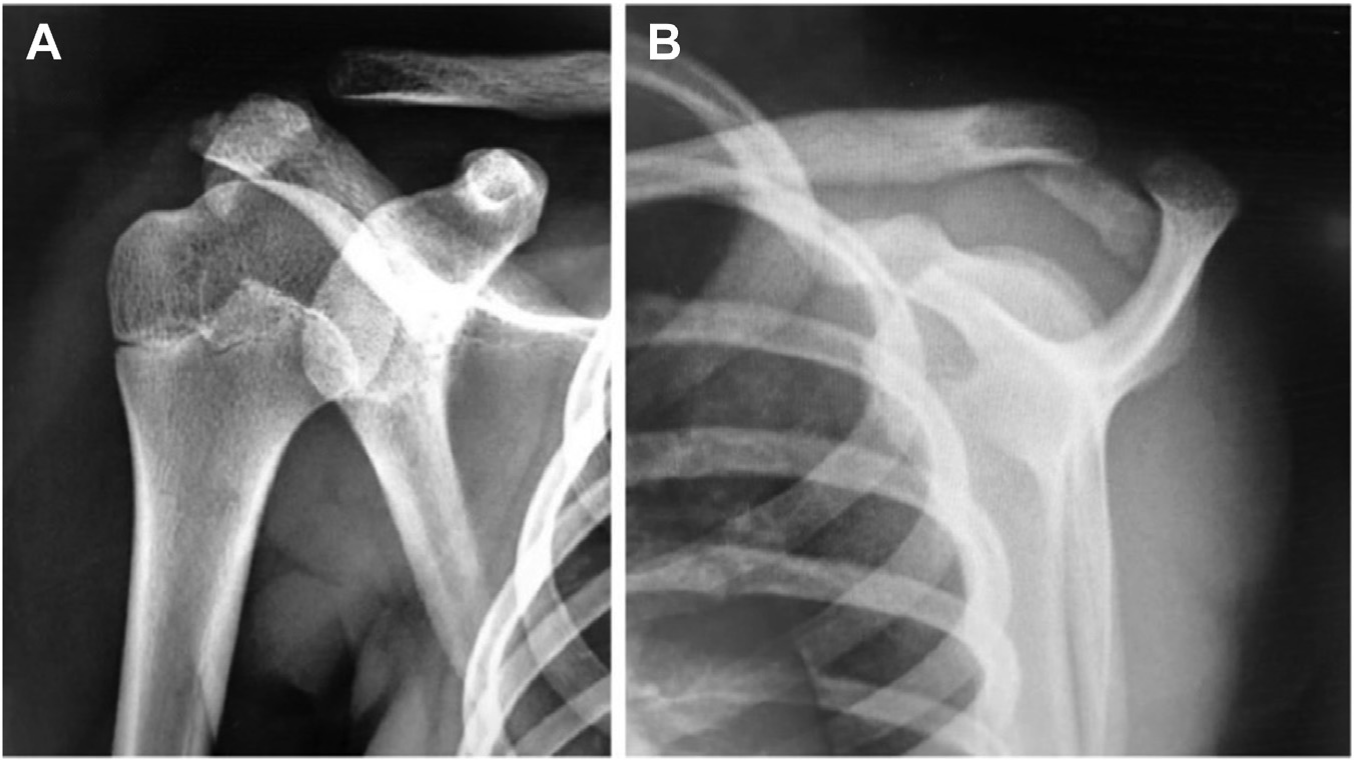
(**A**) Shoulder anteroposterior and (**B**) scapular Y radiographs, showing a displaced bony fragment from the acromion, occupying the subacromial space.

**Figure 2:**
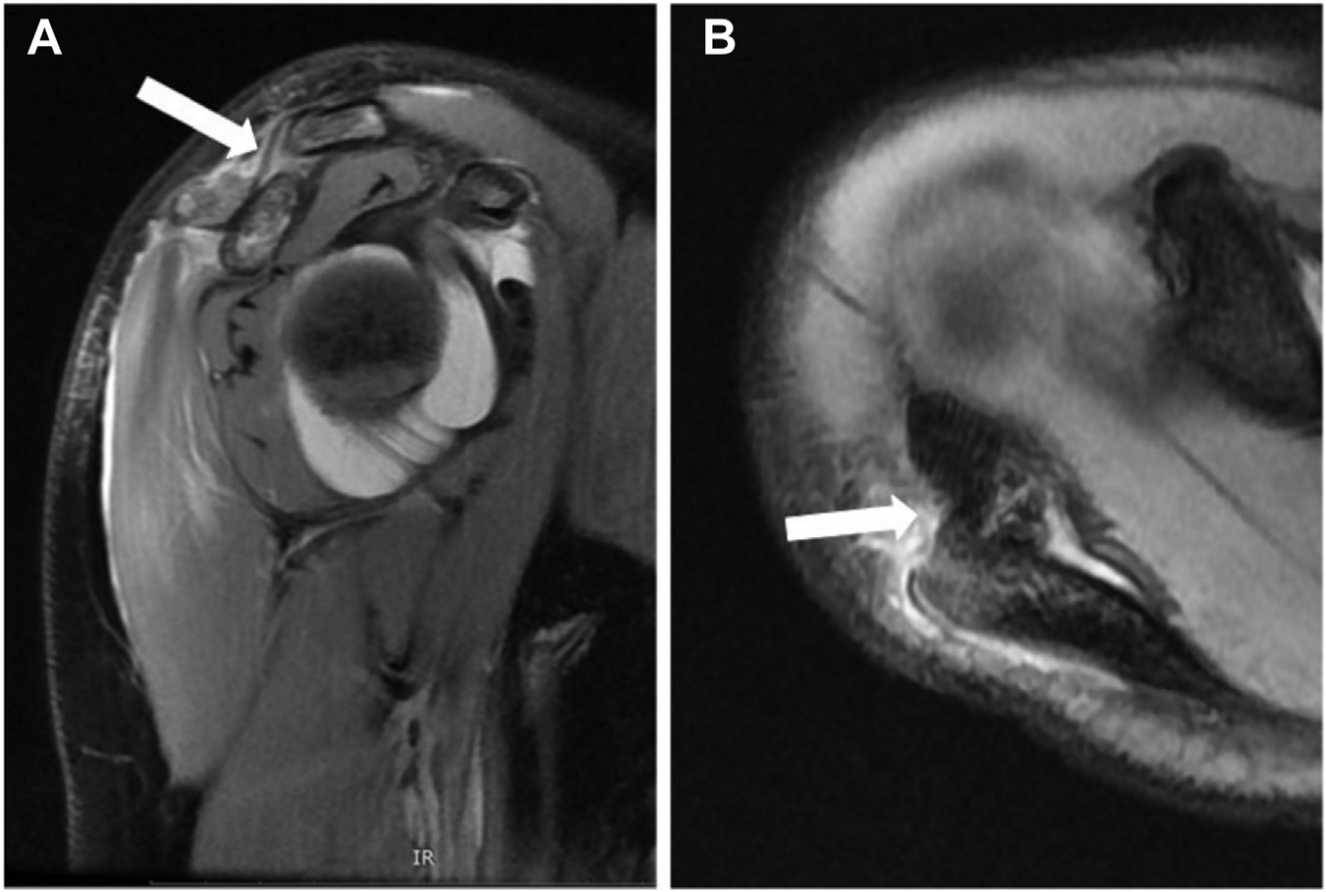
(**A**) Preoperative sagittal plane MRI showing an inferior displaced type-I Salter-Harris physeal fracture (arrow) with subacromial space occupation. (**B**) Preoperative axial plane MRI showing anterior displacement of the acromial fragment (arrow). *MRI*, magnetic resonance imaging.

A 13-year-old girl presented with right acromion pain following a direct fall on her right shoulder while playing volleyball. Physical examination revealed pain at the acromion, with limited shoulder motion, particularly in flexion and abduction. The acromioclavicular joint (ACJ) was stable and neurovascular assessment was normal. Shoulder anteroposterior and scapular Y radiographs showed a displaced bony fragment from the acromion, occupying the subacromial space (Fig. 1). Further assessment with MRI confirmed an isolated anteroinferior displaced type-I Salter-Harris physeal fracture with subacromial space occupation (Fig. 2).

**Figure 3:**
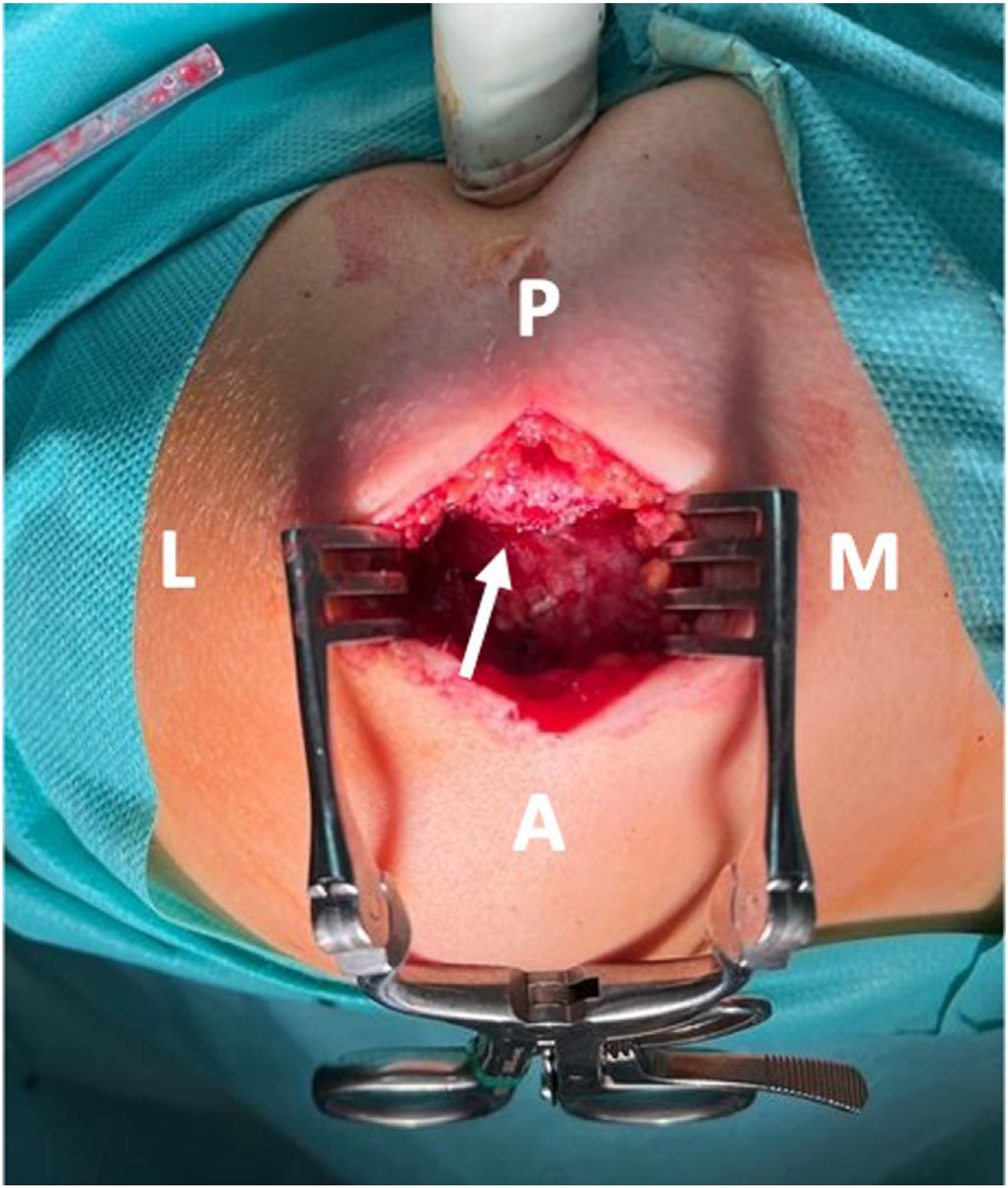
Superior view of the acromion revealing an anteroinferior displaced meso-acromion physeal injury (arrow). *P*, posterior; *M*, medial; *A*, anterior; *L*, lateral.

**Figure 4:**
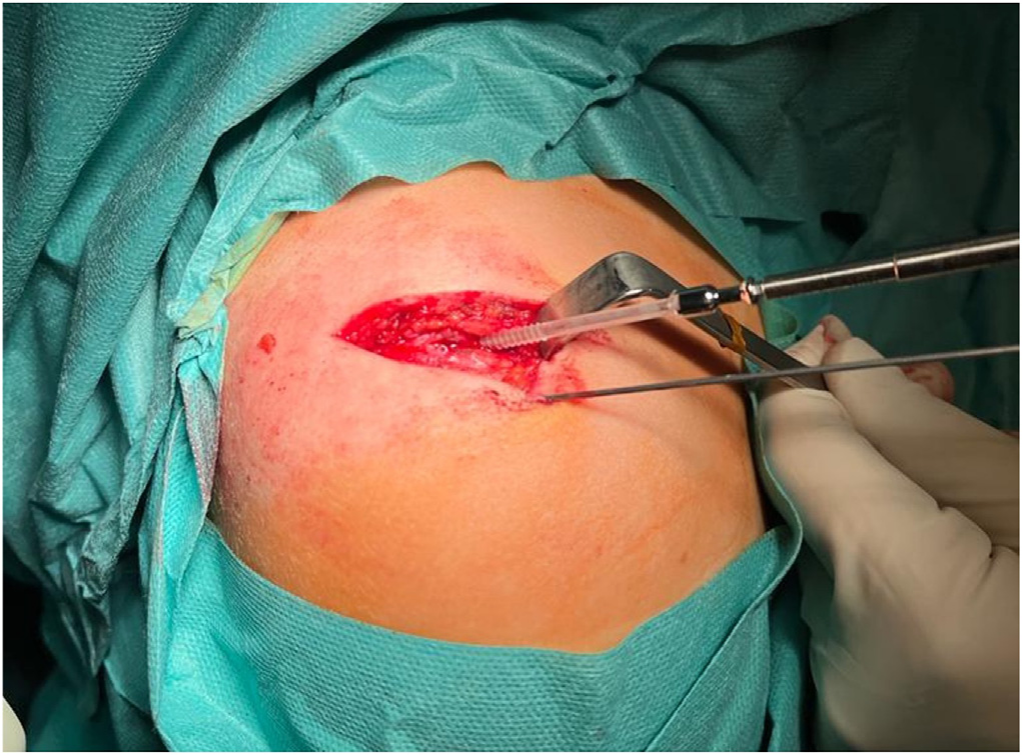
Superolateral view of surgical fracture fixation with 2 parallels anteroposterior K-wire-guided 3.5-mm cannulated resorbable poly lactic-co-glycolic acid screws.

The shoulder was immobilized in a sling and surgery was performed 2 days after the injury when the operating room became available. A direct open approach to the acromion was carried out, revealing an anteroinferior displaced meso-acromion physeal injury (Fig. 3). A careful open reduction was performed using a curved bone chisel. Temporary fixation was achieved with 2 parallel K-wires, and the quality of the reduction was assessed with the C-arm. Fixation was then achieved with 2 parallel anteroposterior K-wire–guided 3.5-mm cannulated resorbable poly lactic-co-glycolic acid (PLGA) screws (ActivaScrew; Bioretec, Tampere, Finland) (Fig. 4). Postoperative radiographs showed a satisfactory reduction of the fracture.

The shoulder remained immobilized in a sling for 1 week. Pendulum movements were allowed immediately after surgery. Active motion was gradually allowed, starting from the second postoperative week, while return to sports was restricted until 3 months after the surgery.

**Figure 5:**
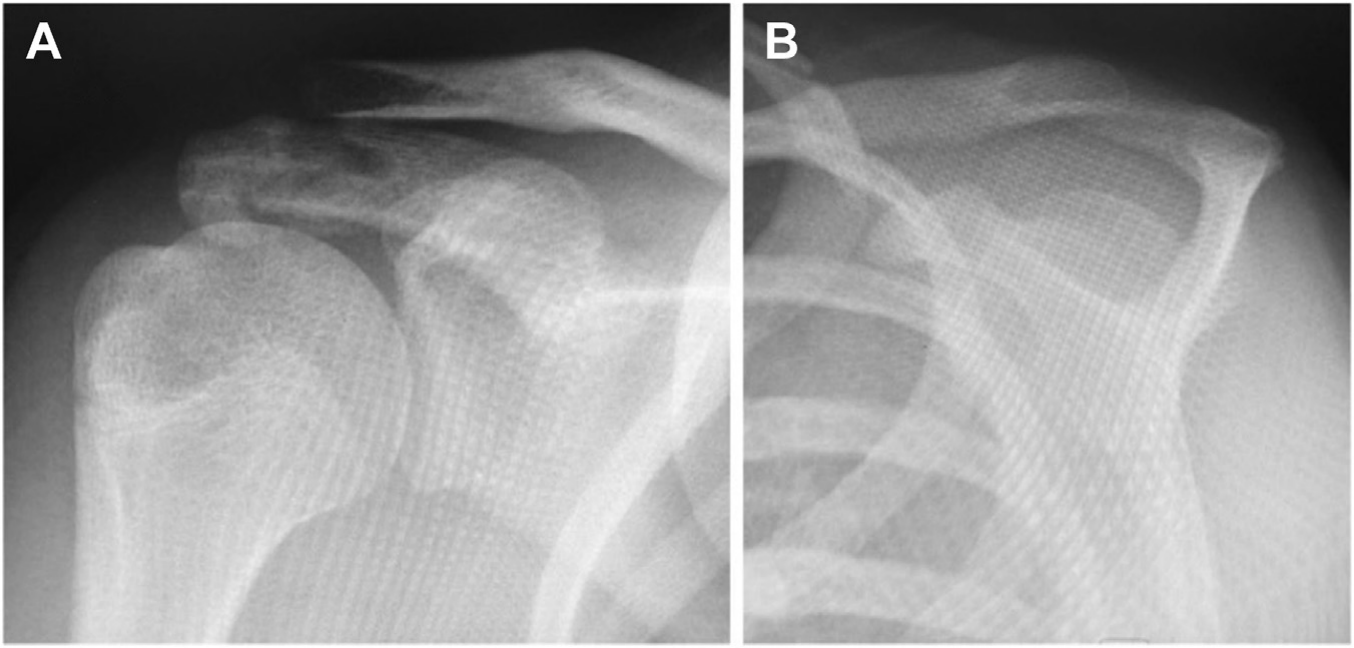
(**A**) Three-month postoperative shoulder anteroposterior and (**B**) scapular Y radiographs showing fracture healing and bone bridging between the epiphysis and metaphysis.

**Figure 6:**
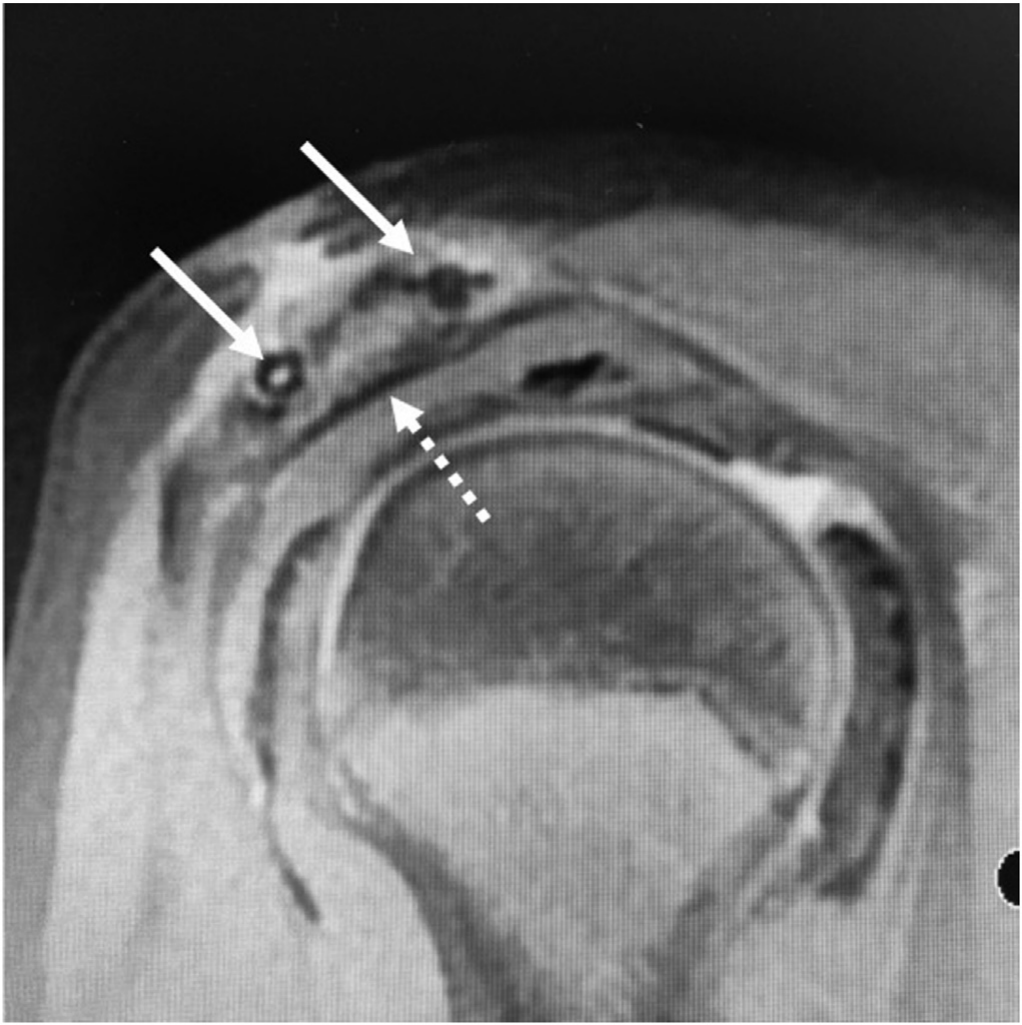
Three-month postoperative coronal plane MRI, showing well-positioned screws and physeal ossification (arrows) with bone bridging between the epiphysis and metaphysis (dotted arrow). *MRI*, magnetic resonance imaging.

Follow-up visits were scheduled at 2 weeks, 6 weeks, 3 months, 6 months, and 1 year after the surgery. At each visit, radiograph control was performed, and a postoperative MRI was conducted at the 3-month visit, revealing well-positioned screws and physeal ossification with bone bridging between the epiphysis and metaphysis (Figs. 5 and 6). At the 6-month follow-up, the patient had completely recovered clinically and functionally, with a full range of shoulder motion and adequate wound healing. The Visual Analog Scale rated 0 mm, and the Nottingham Clavicle Score was excellent, scoring 96 points. At the 1-year follow-up, the patient showed no functional or clinical impairment, and there were no signs of local growth discrepancy in the clinical or radiological evaluation.

## Discussion

Acromion fractures are uncommon, constituting less than 8% of all scapular fractures in adults. They are often associated with other injuries, such as clavicle fractures, coracoid fractures, or ACJ injuries.^10^ The most common cause of these fractures is a direct fall onto the shoulder.^11^ In pediatric populations, this mechanism often leads to distal clavicle fractures in children aged less than 13 years and ACJ dislocations in children aged 13 years or more, making isolated acromion fractures extremely rare.^4^ Nonetheless, scarce cases of acromion fractures in children have been reported.^1^^,^^2^^,^^8^^,^^12^ Among these cases, 2 were attributed to myoclonus seizures, with one infant suffering from tetanus neonatorum and the other from malignant osteopetrosis.^1^^,^^8^ Additionally, acromion fractures have been reported as incidental findings during skeletal surveys conducted when child abuse syndrome was suspected.^2^

Physical examination in infants can be challenging, and certain signs such as irritability, limited arm movement, and unexplained injuries like bruises or fractures inconsistent with the child’s developmental ability may raise suspicion of child abuse syndrome.^2^ In older children and adolescents, localized pain and impaired shoulder function are commonly observed. Although accurately assessing shoulder range of motion can be difficult due to pain, attempts should be made to evaluate potential impingement or mechanical motion blockage. Furthermore, palpation of the distal clavicle and ACJ, as well as ACJ stability assessment, is essential to rule out associated injuries like clavicle fractures or ACJ dislocations.

Radiographs are typically used to confirm the diagnosis, but additional imaging studies should be obtained to ensure an accurate assessment of the injury. In pediatric cases, MRI is particularly valuable in confirming and evaluating physeal injuries, as well as identifying any accompanying injuries, while minimizing exposure to radiation.^14^

**Figure 7:**
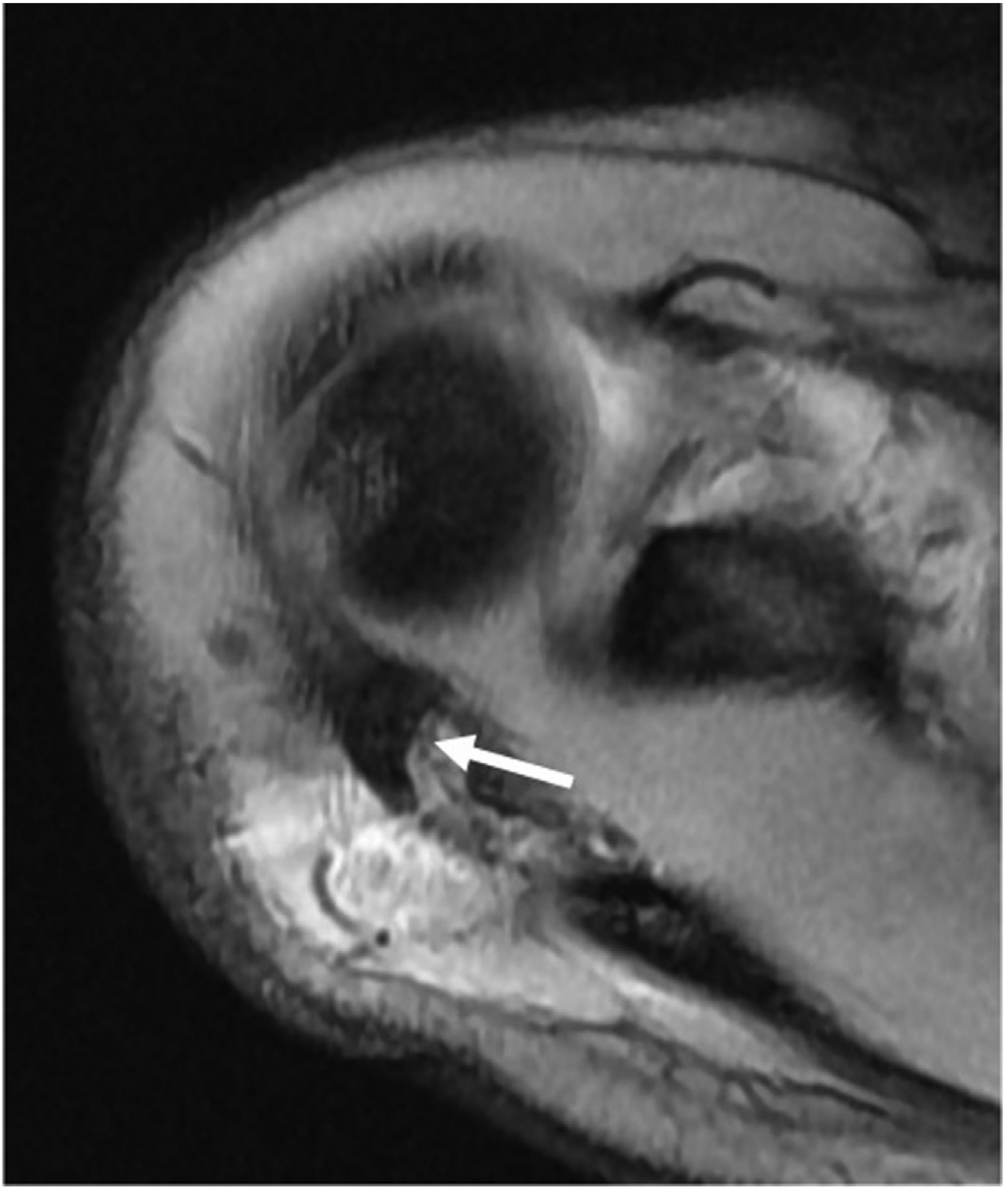
Preoperative axial plane MRI showing a displaced meso-acromion ossification center with a posteriorly curved physis (arrow). *MRI*, magnetic resonance imaging.

Os acromiale, a condition resulting from the failure of fusion of any of the secondary ossification centers of the acromion, can often mimic acromion fractures, making the differential diagnosis challenging. Three distinct secondary ossification centers exist in the acromion: pre-acromion, meso-acromion, and meta-acromion, in a distal to proximal arrangement.^14^ Axial MRI can help differentiate normal ossification centers from an os acromiale. A normal physis typically exhibits a curved extension along the lateral aspect of the acromion, while an os acromiale is characterized by a transverse interference line, separating it from the acromion.^14^ It is essential to note that an os acromiale diagnosis should be reserved for adults, as ossification centers are expected to fuse by the age of 18-25 years.^5^ However, if a transverse interference line is observed on axial MRI in an adolescent, this may preclude the diagnosis of an os acromiale. This aligns with our case, where the axial MRI revealed a displaced meso-acromion ossification center with a posteriorly curved physis, consistent with a type-I Salter-Harris fracture (Fig. 7).

Nondisplaced or minimally displaced fractures can be managed nonoperatively. However, displaced fractures, especially those occupying the subacromial space, often require surgical treatment to prevent complications like subacromial impingement and rotator cuff tears, which can result in shoulder weakness, restricted motion, and pain.^9^

Various surgical techniques, such as plates, K-wires, tension band wiring, cannulated screws, and fragment excision for very small fragments, have been described for treating acromion fractures.^12^ In this particular case, we opted for cannulated screws due to their ability to achieve stable fixation in the absence of comminution and to avoid complications associated with wires, such as migration and the need for removal. Additionally, we selected resorbable PLGA screws to minimize the need for future osteosynthesis removal surgeries, which are often required in children with nonresorbable implants. Resorbable implants in children have shown satisfactory bone healing without causing growth arrests. However, it is important to mention some drawbacks of these devices, such as their radiotransparency, which makes it challenging to assess their position or implant displacement using radiographs. On the other hand, this property becomes advantageous for MRI due to reduced artifact interference.^13^ The most significant complication associated with these implants is local inflammatory reactions during the bio-absorption process, which has been reported in the literature.^7^ Most of these reactions are linked to the use of first-generation resorbable implants made of poly-lactic acid, which take years to fully reabsorb.^6^ In contrast, PLGA implants in children exhibit a faster bio-absorption rate, possibly due to the continuous growth of bone and a higher metabolic rate, leading to reduced local inflammatory complications.^7^ Although our patient did not experience any local inflammatory reaction during the 1-year follow-up, in vivo animal models have shown that resorption occurs within 18 months.^7^ Thus, it is advisable to consider a minimum of a 2-year follow-up period for patients who have received resorbable implants.

Functional and radiological results reported in the literature demonstrate satisfactory outcomes for acromion fractures treated surgically.^3^^,^^12^ In our patient’s case, a full shoulder range of motion was regained, and the patient returned to her previous level of sport activity without any pain at the 6-month follow-up. We used the Nottingham Clavicle Score to assess the ACJ-specific patient-related outcome, and while an excellent outcome was achieved, perfection was not attained due to patient’s aesthetic scar concerns.

## Conclusion

Isolated physeal acromial injuries are extremely rare. MRI is essential for precise evaluation and treatment planning. Surgery must be indicated in displaced injuries, especially those occupying the subacromial space. Resorbable implants are promising fixation options in pediatric fractures with low-rate local complication rates, although extended follow-up should be considered when using them.

## Disclaimers:

Funding: No funding was disclosed by the authors.

Conflicts of interest: The authors, their immediate families, and any research foundation with which they are affiliated have not received any financial payments or other benefits from any commercial entity related to the subject of this article.

Patient consent: Consent was obtained from the patient and her parents that data concerning the case would be submitted for publication.
